# A Compact Imaging Platform for Conducting *C. elegans* Phenotypic Assays on Earth and in Spaceflight

**DOI:** 10.3390/life13010200

**Published:** 2023-01-10

**Authors:** Taslim Anupom, Siva A. Vanapalli

**Affiliations:** 1 Electrical Engineering, Texas Tech University, Lubbock, TX 79409, USA; 2 Chemical Engineering, Texas Tech University, Lubbock, TX 79409, USA

**Keywords:** *C. elegans*, phenotyping, microfluidics, spaceflight imaging

## Abstract

The model organism *Caenorhabditis elegans* is used in a variety of applications ranging from fundamental biological studies, to drug screening, to disease modeling, and to space-biology investigations. These applications rely on conducting whole-organism phenotypic assays involving animal behavior and locomotion. In this study, we report a 3D printed compact imaging platform (CIP) that is integrated with a smart-device camera for the whole-organism phenotyping of *C. elegans*. The CIP has no external optical elements and does not require mechanical focusing, simplifying the optical configuration. The small footprint of the system powered with a standard USB provides capabilities ranging from plug-and-play, to parallel operation, and to housing it in incubators for temperature control. We demonstrate on Earth the compatibility of the CIP with different *C. elegans* substrates, including agar plates, liquid droplets on glass slides and microfluidic chips. We validate the system with behavioral and thrashing assays and show that the phenotypic readouts are in good agreement with the literature data. We conduct a pilot study with mutants and show that the phenotypic data collected from the CIP distinguishes these mutants. Finally, we discuss how the simplicity and versatility offered by CIP makes it amenable to future *C. elegans* investigations on the International Space Station, where science experiments are constrained by system size, payload weight and crew time. Overall, the compactness, portability and ease-of-use makes the CIP desirable for research and educational outreach applications on Earth and in space.

## 1. Introduction

Whole-organism phenotypic assays are a foundational tool for basic biology investigations, drug screening and disease-focused studies [1,2,3,4]. The nematode *Caenorhabditis elegans* is particularly relevant for phenotypic screening due to its small size (≈1 mm), fast reproductive cycle (≈3 days), short lifespan (≈3 weeks), amenability to genetic manipulation and significant translational relevance [5,6,7]. Its small size and ease of culture offer the ability to phenotype large populations offering statistical power, which can be a challenge with vertebrate models. Whole-organism phenotypic readouts in *C. elegans* typically involve examining animal behavior, locomotory prowess and survival in response to changes in environment [8,9,10,11,12,13,14,15].

The numerous advantages that underscore the use of *C. elegans* as a genetic model on Earth also translate to its use in spaceflight as a cost-effective organism to understand the mechanisms underlying the effects of microgravity and radiation exposure on astronaut health. Indeed, *C. elegans* have been flown several times to the International Space Station (ISS) [16,17,18,19,20]. The conducting of animal experiments on the ISS is associated with stringent operational requirements on payload size, weight, crew time and safety [21,22]. *C. elegans* offers a facile means to address these requirements, making it an efficient model for space-biology investigations.

Standard microscopes have been the workhorse for image-based whole-organism phenotyping in *C. elegans*. The need to increase assay throughout and reduce costs has led to the development of a variety of bespoke imaging solutions. Approaches range from the use of scanners for parallel imaging of nematodes on agar plates [23,24] to compact do-it-yourself (DIY) systems based on Raspberry Pi cameras [25,26,27] and smartphones [10,28,29]. Scanners offer a convenient means to increase *C. elegans* assay throughout, but are bulky and generate heat that requires significant customization to maintain temperature during the assay [23]. The Raspberry Pi camera approach offers on-board computation, making it attractive; however, it requires an external display for viewing and keyboard, which can be cumbersome, especially for ISS experiments.

The smartphone microscopy approach has promise to create compact, portable *C. elegans* imaging systems with high-resolution displays and on-board computation. Current methods focus on high-magnification and multi-contrast imaging of *C. elegans*, lending to the inclusion of optical and mechanical elements [10,26,28,29]. These elements increase the complexity of the optical configuration and system size/weight. Considerations on power requirements, parallel experimentation and assay temperature control have received less attention, which are essential for spaceflight studies. Since *C. elegans* assays are conducted in a variety of substrates, compatibility with these substrates will broaden the utility of such compact systems. Finally, thorough validation and mutant phenotypic data needs to be generated from smartphone microscopy studies that will lower barriers for adoption in research and educational settings.

In this study, we report a small foot-print smart-device imaging system called Compact Imaging Platform (CIP) that can acquire videos of *C. elegans* in agar plates, liquid droplets on a glass slide and microfluidic devices. The system has no external optical or mechanical elements, thereby reducing the complexity for imaging. The CIP is powered by USB and can be housed in standard incubators offering a plug-and-play approach for science investigations on Earth and in space. We conducted validation and mutant studies on Earth to show the promise of CIP for *C. elegans* phenotypic assays.

## 2. Results and Discussion

### 2.1. Description of the Compact Imaging Platform and Its Components

**Figure 1:**
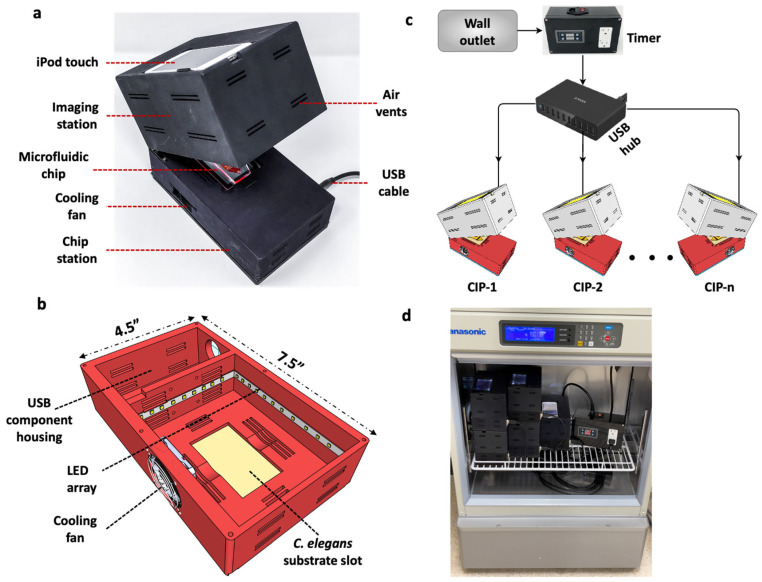
Design of the Compact Imaging Platform and its functional capabilities. (**a**) The CIP consists of an imaging station to hold iPod Touch for imaging, a *C. elegans* substrate and a cooling fan for temperature control. It is powered by a 5 V USB cable. (**b**) The bottom section of the CIP showing the LED array and the USB component housing. (**c**) Multiple CIP units powered with a USB hub with the illumination turned on/off using a programmable timer. (**d**) Five CIP units stacked, operated in parallel and placed in an incubator with the timer.

Design considerations such as light weight, compactness and low power consumption are not only relevant for Earth-based *C. elegans* applications, but become essential for spaceflight studies. For example, the ISS is a closed environment with limited workspace for conducting experimental investigations; moreover, payload size and weight become crucial factors dictating whether an experiment should be launched. Considering these needs, we designed and built a compact imaging platform (CIP). As shown in Figure 1, CIP is a small unit with length L = 7.5″, width W = 4.5″ and height, H = 5.5″, occupying a footprint area of ≈34 in^2^ and weighing ≈ 700 g. The footprint is primarily determined by the need to accommodate the smartphone system, a fan for temperature control and the *C. elegans* substrate.

The mechanical design of the CIP is simple. It consists of two sections made from Polyamide 12 nylon material and printed using a multi-jet fusion 3D printer (HUBS, Chicago, IL, USA). The two sections are connected by hinges that allows their opening and closing (Figure 1a). The top section houses the iPod Touch camera (Apple Inc, Cupertino, CA, USA), and the bottom section houses the *C. elegans* substrate, 6500 k pure white LED lights (Power Practical Inc., Salt Lake City, UT, USA) and a 6000-rpm cooling fan (Wathai, China) (Figure 1b). Thin rectangular slots of 20 mm × 2 mm are provided on the top and bottom sections, acting as air vents to achieve thermal equilibrium when the CIP is placed in a temperature-controlled environment. The thermal management system of the CIP works independently of the gravitational environment (ground or microgravity), due to active cooling and proper ventilation present in the system.

The imaging configuration of the CIP involves dark-field imaging of *C. elegans* samples by using a fixed-height illumination source and the iPod Touch camera. The white LED lights in the bottom section are secured along the four walls, providing sidewise illumination to the specimen. No external optical elements or mechanical focusing is needed, making the design simple and user friendly. Autofocusing is achieved using a 3rd-party app (FiLMiC Pro, Seattle, WA, USA) on the iPod Touch. This app also allows for adjustment of the magnification, field of view and frame rate of video acquisition. The fixed focal distance configuration gives a field of view of 15 mm × 23 mm to 38 mm × 68 mm depending on the optical 3x zoom available on the app. Videos can be acquired at 1080p resolution with a frame rate of 3–30 fps, or time-lapse imaging can be performed with a delay ranging from 1–60 s.

A smart-device camera such as the iPod Touch was chosen not only because of its ease of use on Earth, but also because of the specific advantages it provides for *C. elegans* experiments on the ISS. Unlike bulky conventional microscopy systems, it is a compact integrated imaging system that not only has optical elements for visualization, but also has a display (rather than a computer monitor) that allows the crew to inspect the animals and image quality. Additionally, the iPod Touch has the capacity to store up to ≈250 GB worth of data, allowing for recording of up to ≈810,000 images which enables short-term (e.g., about 1800 movies of 1.5 min duration at 5 fps) and long-term behavioral assays (e.g., about 45 h at 5 fps). Image acquisition aspects such as autofocusing and video capture rate can be easily controlled using the installed app. Finally, conducting science experiments with specialized hardware on the ISS requires significant astronaut training; however, the crew is familiar with using smartphone devices and associated apps, making our smart-device approach crew-friendly for spaceflight experiments.

The CIP, integrated with the smart-device camera, is a 3 W (5 V, 0.6 A) low-powered system that can be battery operated if needed. The electrical requirement involves powering the LED lights and the cooling fan. Both elements are powered by a 5 V USB wall charger and a USB Type-A to Type-A cable. We chose this method because battery packs are available that can directly power our CIP using USB cables. This makes the CIP unit portable for field, educational and outer space applications. Thus, the operational simplicity of the CIP makes it attractive, compared to standard microscopes, to pursue phenotypic studies in *C. elegans* on Earth and for spaceflight studies.

A major advantage of the USB-powered CIP unit is that multiple units can be powered simultaneously using a multi-port hub without the need for individual wall chargers (Figure 1c). In addition, a programmable timer can be added that controls illumination intervals for long-term assays. The plug-and-play nature of the CIP and its small footprint allows the parallel operation of several units inside a standard incubator (Figure 1d) with desired temperature control. This new capability allows multiple assays with the same or different strains to be conducted simultaneously in the same substrate or in different substrates (plates, microfluidic chips etc.). The ability to house the CIP in an incubator is also conducive for spaceflight investigations, as the ISS accommodates several incubators. For example, the Space Automated Bioproducts Laboratory (BioServe Space Technologies, Boulder, CO, USA) is a dual-function freezer/incubator that supports life-sciences experiments on the ISS with a temperature control of −5 to 43 °C and accommodates 2 USB ports [30].

### 2.2. Compatibility of the CIP with Different C. elegans Substrates

**Figure 2:**
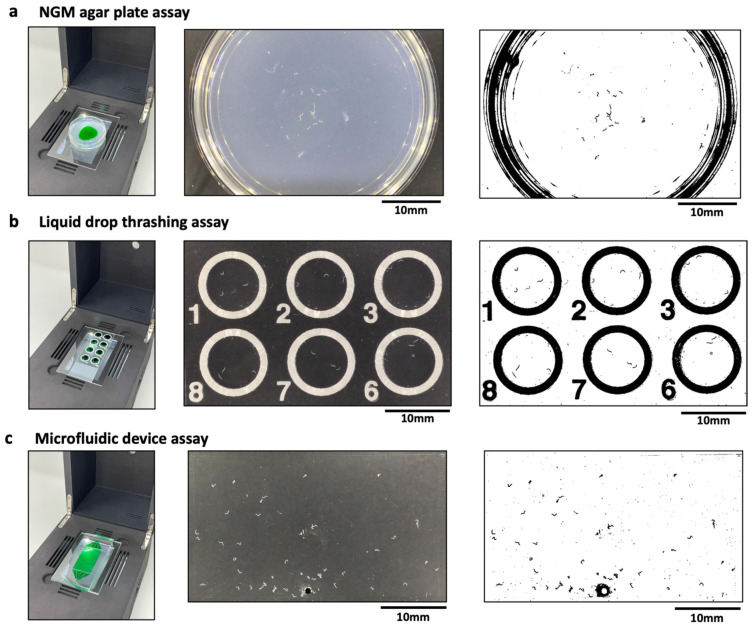
Compatibility of the CIP with various C. elegans substates. (**a**) Imaging of animals crawling on a 3 cm agar plate (**left**). The actual (**middle**) and processed (**right**) image are also shown with *n* = 24 animals. The green dye region indicates the bacterial lawn. (**b**) Imaging of animals thrashing in liquid drops (**left**). The actual (**middle**) and processed (**right**) image are also shown with *n* = 3 animals per drop. The field of view allows imaging of 6 wells. (**c**) Imaging of animals crawling in a microfluidic pillar arena (**left**). The actual (**middle**) and processed (**right**) image are also shown with *n* = 51 animals in the arena. The images were processed using Image J V 1.53 (NIH, Maryland, USA), wrMTrck plugin.

Phenotypic assays in *C. elegans* often involve imaging animals placed on a variety of substrates, ranging from agar plates to liquid drops on glass slides to microfluidic devices. Here, we imaged wild-type animals crawling on agar plates, which is a translucent medium of thickness ≈ 5 mm (Figure 2a); animals swimming in sessile liquid drops of peak height ≈ 2 mm that partially wet a glass-slide (Figure 2b); and animals crawling in a microfluidic pillar chip that is filled with a liquid (Figure 2c) [12]. Each of these substrates have different refractive indices, optical path lengths and interfaces, which can alter the transmission of light and therefore can affect image contrast. Versatile imaging systems need to be capable of visualizing animals in these different substrates. We therefore tested the ability of the CIP to obtain sufficient-quality images when adult animals are housed in different substrates.

An important aspect of image-based assays in *C. elegans* is the analysis of images to quantify phenotypic readouts. Standard image processing techniques, for example, based on thresholding the intensity of the target objects against background intensity, work best when there is sufficient intensity contrast between the object and the background, and the objects occupy enough pixels for successful detection. Figure 2 shows representative images obtained from the three substrates using the CIP. We find that the images from these substrates are of sufficient quality such that the adult animals can be easily segmented using standard image processing software (ImageJ [31,32], wrMTrck plugin [33]). Under optimal imaging conditions, we find day 1 gravid adults to occupy ≈50 pixels when housed in the three substrates. This resolution allows these animals to be segmented easily against the background. Because of the field of view and the types of substrates, we find that the CIP is well suited to obtain phenotypic data from ≈20–50 animals, with the large-arena microfluidic chip providing data on more animals.

### 2.3. Controlling CIP Temperature during C. elegans Assays

*C. elegans’* behavior and survival is sensitive to temperature [34,35,36]. In addition, assays purposefully expose animals to different temperatures to investigate genes or compounds that impact thermotolerance [37,38]. Thus, *C. elegans* assays necessitate control over the environmental temperature. Often, the agar plates or microfluidic devices are kept in a temperature-controlled incubator and taken out for imaging on standard microscopes at room temperature. Alternatively, the temperature of these *C. elegans* substrates are controlled by passive or active thermal management strategies. Scanners use fans to control temperature [23], while some studies have used Peltier elements integrated with microfluidic devices assays [39].

The small size of the CIP system allows it to be placed in a temperature-controlled incubator (Figure 1d). The design of the CIP makes it an enclosed system housing the *C. elegans* substrates, since the top section (containing the smart-device camera) is brought in contact with the bottom section during imaging. The Initial trials showed that the main internal source of heat that the *C. elegans* substrates experience in the CIP is the radiant energy from the array of LED lights in the bottom section, which leads to an increase in temperature. To address this issue, we created ventilation slots in the enclosure and installed a cooling fan at the left wall of the bottom section. The forced convection from the cooling fan draws air from the ambient and distributes it through the ventilation slots.

**Figure 3:**
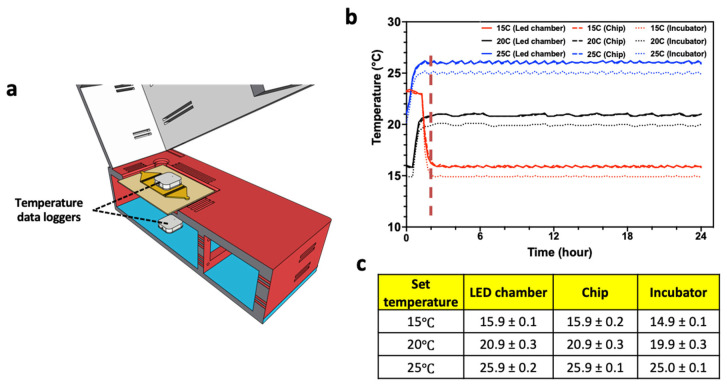
Controlling temperature of CIP. (**a**) Temperature was measured at the top of the microfluidic chip and in the LED chamber. (**b**) Temperature profile collected over 24 h at set incubator temperatures of 15 °C, 20 °C, and 25 °C. The temperature data of LED chamber and the microfluidic chip are nearly identical. (**c**) Table showing mean temperature and standard deviation from all the three collection points.

To test the ability to control the temperature of the *C. elegans* substrate housed in the CIP, we conducted experiments in which the system was placed in the incubator along with two temperature data loggers—one in the LED chamber and the other on the microfluidic chip (Figure 3a). This experiment was conducted for three set temperatures of 15 °C, 20 °C, and 25 °C of the incubator, and measurements were taken for 24 h. As shown in Figure 3b,c, in less than 2 h, the temperature stabilizes for all three set temperature conditions, with a variation that is less than 0.5 °C. We also note that the CIP temperature is about 1 °C above the set temperature, due to local heating, which can be compensated by lowering the incubator temperature. Thus, adequate temperature control can be achieved for *C. elegans* assays using the CIP.

### 2.4. Validation of the CIP with Behavioral Assays on Different C. elegans Substrates

**Figure 4:**
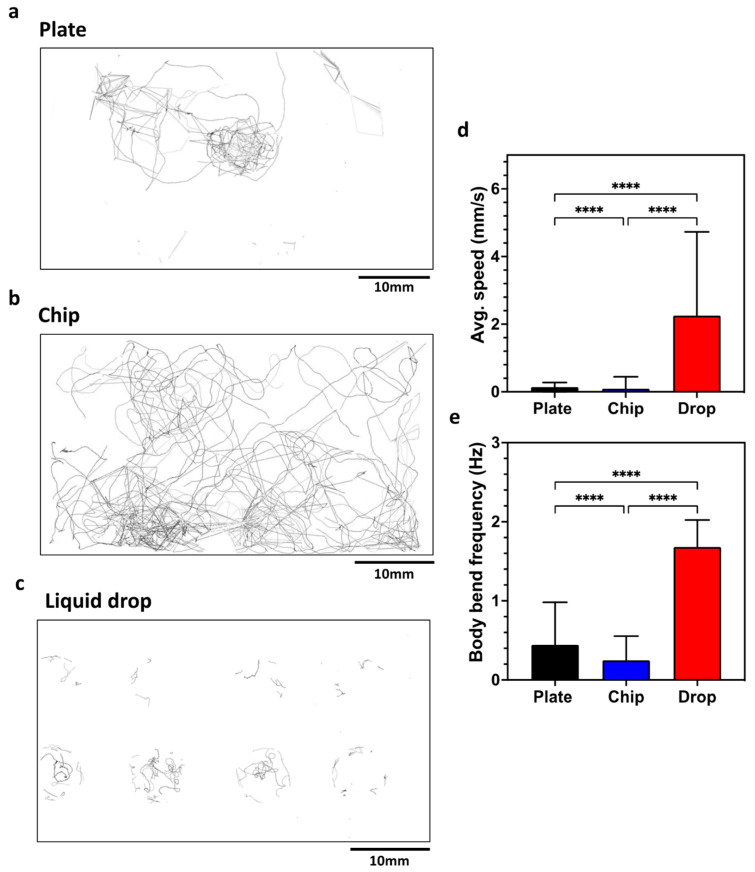
Validation of the CIP using *C. elegans* behavioral assays on a plate, a chip and liquid drops. (**a**–**c**) Overlay of tracks made by animals in the three substrates. (**d**) Average speed of animals on the three substrates. Plate, *n* = 24; chip, *n* = 51, *p*-value ≤ 0.0001; drop, *n* = 24, *p*-value < 0.0001. Animal age is day 5 from hatching. (**e**) Body bend frequency of animals on the three substrates. Plate, *n* = 24; chip, *n* = 51, *p*-value ≤ 0.0001; drop, *n* = 24, *p*-value < 0.0001. Animal age is day 5 from hatching; alpha = 0.05 for all analysis, nonparametric Kruskal–Wallis test.

The CIP is a simple-to-use phenotyping system for *C. elegans* assays. In this section, we validate the system by performing standard behavioral assays on plates, liquid drops and microfluidic chips. We initially tested wild-type animals on Nematode Growth Media (NGM) plates to quantitate the locomotory readouts (Figure 4) obtained from the CIP and compare them with the data reported in literature. The CIP set up was similar to that shown in Figure 2a. Videos were recorded for 5 min at 10 fps, and data were analyzed with the wrMTrck plugin in ImageJ. Figure 4a shows the trajectory overlay that provides a qualitative visualization of the animal behavior on plates (Refer to SI Video 1 for processed video). We extracted the average forward speed and body wave frequency, and found the values to be ≈0.14 mm/s and ≈0.39 Hz (Figure 4d,e). These values are close to the reported values of ≈0.20 mm/s and ≈0.30 Hz in the literature [40,41].

Next, we tested the same animals in microfluidic chips that have an array of micropillars arranged in a square lattice, with a gap of 90 μm between pillars. Videos were recorded for 5 min at 10 fps. Figure 4b shows the trajectory overlay of animals crawling in the microfluidic chip (Refer to SI Video 2 for processed video). The average forward speed and body wave frequency were found to be ≈0.10 mm/s and ≈0.25 Hz (Figure 4d,e). In pillar arenas, the forward speed and body frequency depend on the pillar lattice arrangement and the animal confinement between the pillars [15,42,43]. Our locomotory data from the CIP is in good agreement with that of Rahman et al. [14], who reported values of ≈0.13 mm/s and ≈0.21 Hz.

We also tested the same animals in a liquid drop on a glass slide with eight Teflon wells 8 mm in diameter. We transferred three animals in 25 µL of NGM liquid. Videos were recorded for 5 min at 10 fps. Figure 4c shows the trajectory of the overlay of animals swimming in the liquid drop (Refer to SI Video 3 for processed video). The average forward speed and body wave frequency were found to be ≈2.30 mm/s and ≈1.68 Hz (Figure 4d,e). This swim data are close to the reported values of ≈2.71 mm/s and ≈1.76 Hz in the literature [40].

### 2.5. Phenotypic Studies with Mutants

*C. elegans* has been an invaluable model organism for identifying the genetic basis of behavior and locomotion. There are many mutants that have been identified and classified based on automated image analysis, creating a behavioral database [44,45]. Since the CIP can accommodate a variety of *C. elegans* substrates, it could be a useful tool to perform phenotypic studies with mutants. Here, we conduct a pilot study with a wild type and two mutants—*unc-32(e189)* and *unc-79(e1068)*. Hierarchical clustering showed that these two mutants exist in a different phenotypic space in the behavioral database [45], thus forming a testbed to evaluate the capabilities of the CIP.

The *unc-32* gene encodes the alpha subunit of a vacuolar ATP-ase (v-ATPase), an ATP-dependent proton pump which functions to regulate neurotransmitter trafficking through synaptic vesicles and cell acidification [46]. The *unc-32(e189)* mutation results in a reverse ventral coiling phenotype in the posterior region of the animals. Likewise, the *unc-79* gene encodes a large cytosolic protein that is thought to regulate the post transcriptional processing of key voltage-gated sodium and calcium channels [47,48]. The *unc-79(e1068)* loss-of-function mutation results in a “fainting” phenotype that presents as repeating episodes of short bursts of normal movement that abruptly, but momentarily, stops [49]. Such *unc-79* mutants have also shown to be defective in their ability to transition from crawling to swimming [50], a subtle behavioral phenotype that was previously undetectable using classical behavioral metrics. The *unc-32(e189)* was previously characterized using plate-based methods and has not been assessed in any other environment. We conducted thrashing and behavioral assays with the wild type and mutant animals to determine whether these assays, conducted with the CIP, could identify phenotypic differences.

**Figure 5:**
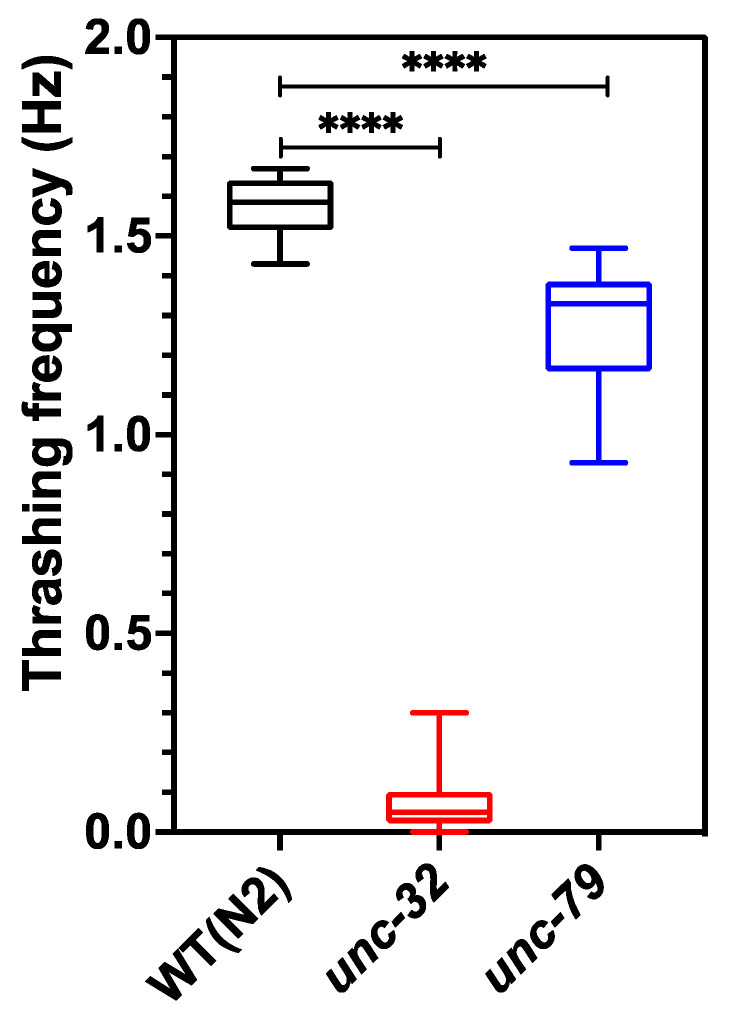
Thrashing assay performed on day 5 animals using CIP. Wild-type animals showed significant difference compared to both *unc-79* (*p*-value < 0.0001) and *unc-32* (*p*-value < 0.0001) mutants. *n* = 10 animals.; one-way ANOVA.

Figure 5 shows the thrashing frequency for the three strains, which was scored manually from the 1 min videos acquired from the CIP at 10 fps. The thrashing frequency of wild type, *unc-32(e189)* and *unc-79(e1068)* is ≈1.57, 0.07 and 1.29, respectively, indicating that mutants have swim defects compared to wildtype, and *unc-32(e189)* has the strongest swim deficiency. The value of the thrashing frequency for *unc-79(e1068)* obtained from the CIP is in good agreement with that found in literature [50,51].

**Figure 6:**
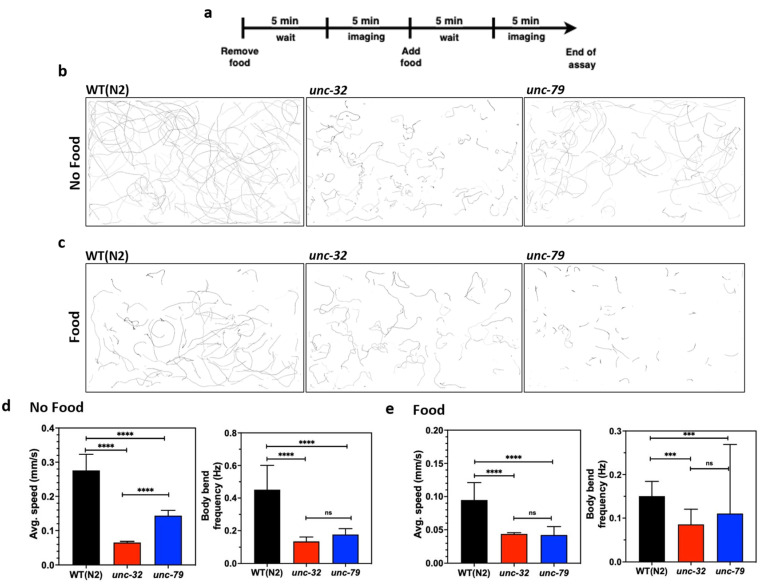
Phenotypic studies with mutants using CIP. (**a**) Protocol used for conducting behavioral assays in the absence and presence of food using day 5 animals. Images were taken 5 min after washing and feeding to allow for acclimatization from the change in environment. (**b**,**c**) Overlay of animal tracks of wild-type and mutants in the absence and presence of food. (**d**) Quantification of average speed and body bend frequency of animal in the absence of food. Wildtype, *n* = 155; *unc-32(e189)*, *n* = 200, *p*-value < 0.0001 (avg. speed), *p*-value < 0.0001 (body bend); *unc-79(e1068)*, *n* =175, *p*-value < 0.0001 (avg. speed), *p*-value < 0.0001 (body bend). (**e**) Quantification of average speed and body bend frequency of animal in the presence of food. Wildtype, *n* = 155; *unc-32(e189)*, *n* = 200, *p*-value < 0.0001 (avg. speed), *p*-value = 0.0002 (body bend); *unc-79(e1068)*, *n* =175, *p*-value < 0.0001 (avg. speed), *p*-value = 0.0.002 (body bend); alpha = 0.05 for all analysis, nonparametric Kruskal–Wallis test.

Next, we studied the crawling behavior of these strains in the microfluidic chip. Since the microfluidic approach allows for the convenient removal and addition of reagents [12,13,52], we studied changes in animal behavior in the presence and absence of food. To perform this assay, day-4 animals were loaded into the chip, and on day 5 the food was removed, followed by a 5-min wait time before imaging (Figure 6a). Subsequently, food was added and imaging was performed after 5 min. The crawling tracks in Figure 6b,c visually reveal not only individual strain differences, but also show food-induced changes in behavior.

From the acquired videos, we quantified changes in the average forward speed and body wave frequency (Figure 6d,e). In the absence of food, the average forward speed of the mutants is statistically different from wildtype (Figure 6d, left) with *unc-32(e189)* being slower than *unc-79(e1068)*. The body wave frequency of wildtype animals is higher than the mutants, but there is no difference between the mutants themselves.

In the presence of food, the average forward speed of the mutants is still statistically different from the wildtype (Figure 6e, left), although the values tend to be lower compared to those in the absence of food. Interestingly, the mutants do not show significant differences in forward speed when food is present. With regards to body wave frequency, there is no apparent difference between the three strains.

Thus, the data from the CIP shows that the thrashing frequency in liquid and the forward crawling speed in the absence of food are better phenotypes to distinguish the strains. These results highlight that the CIP is a versatile tool to conduct mutant phenotyping studies using different *C. elegans* substrates.

### 2.6. Relevance of CIP for C. elegans Spaceflight Studies

Significant physiological changes occur during long-duration space travel, necessitating a fundamental understanding of the mechanisms that induce these alterations [53,54]. Thus, model organisms such as *C. elegans* have been flown to the International Space Station (ISS) several times for space-biology investigations [16,17,18,19,55,56,57,58,59,60]. For spaceflight missions where payload size and weight are critical considerations, *C. elegans* experimental payloads offer a small-form factor and a significant reduction in weight compared with vertebrates. Moreover, the fast reproductive cycle enables multi-generational studies to be conducted, providing an opportunity to explore physiological adaptations due to long-term habitation in space. These advantages and mutant resources make *C. elegans* a leading invertebrate animal model for spaceflight studies.

Despite several *C. elegans* studies on ISS, most of the spaceflight data is limited to changes in gene expression and fluorescent markers, and there is a dearth of whole-organism phenotypic data. The major challenges for ISS investigations are the availability of microscopy hardware, access to crew time to conduct the studies, and payload weight/size requirements for imaging instrumentation. Standard microscopes available on ISS are bulky, require significant crew training, have a limited field of view to do large-population imaging, rely on bulky monitors for crews to visualize samples, and data store/transfer needs are demanding. These limitations necessitate the need for custom-designed imaging hardware for gathering whole-animal phenotypic data to understand the effects of microgravity and radiation on *C. elegans* physiology.

The compact imaging system developed in this study addresses the current limitations for phenotyping *C. elegans* on the ISS. The CIP has <1 kg payload requirement and occupies < 1 ft^3^. It is simple to use for crews and is a USB-powered plug-and-play device. The large field of view enables the interrogation of animal populations and provides a robust statistical evaluation of phenotypic data. Sample visualization and data storage needs are integrated into a small form-factor smart-device. Video data could be returned to Earth, and data analysis could be performed post-facto, thereby allowing the crew time to focus more on worm sample loading and basic payload handling.

Different *C. elegans* substrates can be integrated into the CIP based on science requirements, and the microfluidic chips are particularly appealing due to their enclosed configuration that allows the maintaining of a sterile environment and efficient fluid handling. Nematode sample preparation usually occurs on Earth, with worm culture bags being flown to the ISS, where the crew extracts samples and injects into the microfluidic chips. The authors have recently flown to the ISS micropillar-based microfluidic devices for muscle strength measurements in *C. elegans* [20], and the CIP is a valuable addition to the imaging needs on the ISS.

## 3. Conclusions

In this study, we report a compact imaging platform and successfully demonstrated that it can be used to acquire whole-organism phenotypic data using a variety of *C. elegans* substrates. We show that good temperature control can be achieved for assays, and that multiple CIP units can be used if needed to conduct parallel experiments. The videos acquired are compatible for analysis with established software tools. The *C. elegans* community continues to develop a variety of open-source image analysis tools [61] that could be potentially used to analyze images acquired from the CIP to increase the depth of phenotyping.

The hardware of CIP as demonstrated here could be further improved. In addition to the smart-device camera, additional high-magnification optics could be integrated to provide higher-resolution images to pursue deep phenotyping, including access to organ-level information. Although we used the iPod Touch in this study, the 3D-printed imaging station concept can be extended to other low-cost cameras (e.g., Arducam series), along with Raspberry Pi integration. Opportunities exist to integrate additional hardware elements to study animal response to stimuli such as light, temperature and electric fields, which will broaden the types of assays that can be conducted. With advances in computation and artificial intelligence, apps could be custom-designed to integrate with a smart-device that allows real-time image analysis, storage of assay metadata and data visualization.

In summary, the CIP is a simple, versatile, low-cost and small-footprint phenotyping tool for *C. elegans* assays, that could be deployed in research and educational settings on Earth in its current form. The CIP also presents a proof of concept for future setups for space biology research and could be adapted for ISS-compatible hardware. In the future, the system needs to undergo thorough ISS certification in terms of flammability, materials, off-gassing, electromagnetic interface, etc., as the assessment of multiple factors like safety, system reliability and long-term storage for equipment performance is necessary.

## 4. Materials and Methods

**Worm culture.** Animals were cultured in 60 mm nematode growth medium (NGM) petri plates with 150–200 µL E. *Coli* OP50 at 20 °C. Synchronized animals at day 5 from hatching were used in all the assays performed. For this paper, the strains used were *wild-type* Bristol (N2), CB189 *unc-32(e189) III.*, and CB1068 *unc-79(e1086) III*.

**Plate assay**. Twenty-four age-synced animals were transferred to 35 mm nematode growth medium (NGM) petri plates. The plates were seeded 12 h prior to animal transfer so that the bacteria lawn was fresh and thin to obtain good quality contrast between animals and background.

**Microfluidic chip assay**. Microfluidic devices were fabricated using standard SU-8 soft lithography techniques [62] in polydimethyl(siloxane). The design of the micropillar array is similar to that of Rahman et al., with a pillar diameter of 70 μm, gap of 90 μm and a chamber height of 110 μm. Animals were loaded inside the chip using a 1 mL syringe connected with a 0.032″ ID Tygon tubing (Cole-Parmer, Vernon Hills, IL, USA) with a stainless-steel coupler tube (Instech Labs, FL). Synchronized animals in an NGM plate were flooded with liquid NGM and then transferred to an empty plate. Using a syringe, approximately 65 animals were aspirated from the plate and loaded inside the chip using the loading port. The chip was then flushed with liquid NGM, followed by an addition of 20 mg/mL *E. coli OP50*.

**Thrashing assay**. Synchronized animals in an NGM plate were flooded with liquid NGM and then transferred to an empty plate. Three animals with 25 µL of liquid NGM were aspirated and transferred to each well of the glass slide (Tekdon Incorporated, Myakka City, FL, USA).

**Data analysis.** To analyze the videos acquired from the plate and chip assay, we used the open-source software Image J with the plugin called wrMTrck. For the wildtype movies, thrashing data was also obtained using the wrMTrck plugin. For mutant thrashing assay, we played each movie and manually counted the thrashes. Each assay’s data were initially recorded in Microsoft Excel^®^ (Version is 16.66.1) and then imported to GraphPad Prism software for visualization and statistical analysis. Types of statistical tests used in this study are included at the end of each figure caption.

## Data Availability

All data generated or analyzed during this study are included in this published article.
